# Enhancing Patient Participation in Co-Productive Decision-Making With Personal Value Sets: Clinical Trial Prototype

**DOI:** 10.2196/81623

**Published:** 2026-06-16

**Authors:** Jack Dowie, Mette Kjer Kaltoft, Vije Kumar Rajput

**Affiliations:** 1 Faculty of Public Health and Policy London School of Hygiene & Tropical Medicine London United Kingdom; 2 Clinical Institute University of Southern Denmark Odense Denmark; 3 Faculty of Medicine and Health Sciences Keele Univerisity Newcastle-under-Lyme United Kingdom

**Keywords:** patient preferences, personal utility set, clinical decision support, EQ-5D-5L, patient-reported outcome measures

## Abstract

A new approach has been developed to establish the public value (utility) set for the generic health measure used in quality-adjusted life year estimates. In contrast to conventional approaches, it establishes the complete utility set for an individual and aggregates a sample of these to achieve the public set. The novel way of establishing the complete utility set for an individual has the potential to transform the nature and extent of a patient’s participation in the clinical decision-making process. We have modified the online elicitation of personal utility functions approach to overcome its impracticalities in a clinical consultation. The main modification is the replacement of choice-based items by scale-based ones, on the grounds that the former’s time and cognitive demands, while tolerable in the research context, make it infeasible in practice. The personal utility set for healthcare (PUSH) approach, like the online elicitation of personal utility functions one, may be used with any multidimension, multilevel instrument, including condition-specific ones, but the empirical application here is with the health-related quality of life instrument EQ-5D-5L. PUSH for EQ-5D-5L is a decision support tool in the form of a spreadsheet workbook. The clinician assists nondirectively in the elicitation of the patient’s utility set for EQ-5D-5L. Subsequently, the clinician, drawing on the best available evidence and information, enters the EQ-5D-5L states they judge, on the balance of probabilities, the patient will be in (at an agreed future time point), for specific interventions, plus no intervention. The relevant country’s public set utility for each displayed health state is simultaneously revealed. (Those for 13 countries are in the current template.) It is envisaged that the clinician holds the PUSH template on their computer and opens a new copy for use with each patient. They agree with the patient on what, if anything, is to be saved as part of their electronic medical record. The deliberation following engagement with PUSH and personalized evaluation of the contemplated interventions will typically involve sensitivity testing and possible revision of the patient and clinician inputs. One key responsibility of the clinician is to dispel any “aura of exactness” or pseudo-precision that may be created by the use of precise percentages (or values to 2 decimal places). PUSH participation is to be seen as a component of deliberative co-productive decision-making to which both parties contribute significantly but in role-appropriate ways. The outputs are intended to provide a useful, analysis-framed input into the subsequent discussion and co-produced decision. As a major clinical innovation that transforms both patient participation and clinician contribution, it is advanced here for the discussion and critique that will enable a conceptually sound trial protocol to be developed (including clinician tutoring).

## Introduction

Patient-centeredness is a fundamental ethical principle in any health care service, but it needs to be defined if it is to have operational meaning. We take it to mean that the patient’s participation in the clinical encounter is such that the emergent, co-produced decision reflects their informed preferences regarding the consequences following from each of the available options. Given this definition, we further assume that for most patients, the consequences that matter to them will include the effects of the options on their ability to move around, to undertake self-care (SC) and usual activities (UA), and to avoid pain/discomfort (PD) and anxiety/depression (AD), as well as their impacts on more specific biophysical states and functioning. The former set means that there is a compelling case for the routine use of a generic Health-Related Quality of Life (HRQoL) measure, such as the EQ-5D-5L instrument, in both the descriptive and evaluative aspects of clinical decision-making. Patient-centeredness then requires that the individual patient’s personal value—utility—for each health state should be used, not those of the average member of a relevant group or population (or the clinician’s). The latter are now widely available in the form of public utility sets, developed for use in regulatory-level decisions that affect the clinical availability of some options. While it is important that their personal utilities should be used, we believe the individual patient should have the opportunity to learn how their ones compare with the average, so long as it is not implied that the average has normative significance for them.

It is important to make clear immediately that the case being made here is not for the use of generic measures as patient-reported outcome measures (PROMs). These have become increasingly familiar and found to be acceptable and useful in clinical studies [1]. We are making a very different case for generic measures, based on personal utility sets, forming what may be called “Patient-Reported Input Measures” in a co-productive decision-making process. In other words, to support the ex-ante estimation of the expected effect of different interventions. The ex post effect of an undertaken intervention on the measure (as a patient-reported outcome measure) may be a useful input in a future decision, but not the current one.

We have already hinted at the additional reason for making the average health state utilities in the public set available clinically, alongside the values of the individual. That set (“tariff”) has been developed primarily to support policy decisions taken in pursuit of “cost-effectiveness.” Cost-effectiveness is a second fundamental ethical principle that applies whenever we are in a resource-constrained public health care service. The two ethical principles—patient-centeredness and cost-effectiveness—clash when an intervention that is optimal for this patient involves an “unacceptable” level of net benefits being foregone by other patients in the service. It is then made unavailable clinically on the ethical grounds that it is not “(opportunity) cost-effective.” Different jurisdictions and services determine this ethically unacceptable level in different ways. The most familiar involve interventions being evaluated by their impact on quality-adjusted life years (QALYs), with an incremental cost-effectiveness ratio threshold applied to the result. The QA component of the QALY is assessed in numerous ways, but most use a generic measure of HRQoL such as EQ-5D-5L. Public utility sets for it now exist for many countries, and average utilities are hence readily available for the clinical comparison mentioned above.

The routine clinical use of a generic measure in clinical decision-making has a major bonus wherever cost-effectiveness-based restrictions on clinical provision exist. In some situations, such a restriction will impinge directly on the patient’s decision, most notably by making unavailable the option established to be personally optimal. In this situation, we argue that patient-centeredness requires the patient to have the opportunity to see the main reasons why the legitimate restriction may clash with an evaluation based on their own utilities. This can help indicate the net benefit they are sacrificing for and contributing to the “common good” (of which they themselves are part). If, on the other hand, their utilities are such that the proscribed option emerges as suboptimal for them, they are made aware that no such sacrifice or contribution is involved.

In the second, most common, situation, there is no mandatory cost-effectiveness-based restriction on the options relevant to the patient’s decision. However, almost invariably there will be some guideline or practice recommendations regarding the options. Many take the form of sequential protocols: initial “first line” therapy, “second line” therapy if that is ineffective, and so on. These classifications necessarily involve implicit utility-based trade-offs, reflecting considerations such as relative toxicity and invasiveness as well as cost. If the implicit utility-based trade-offs are supported by formal cost-effectiveness analysis, the argument of the previous paragraph applies. More likely, the embedded preferences are “expert panel-based,” and here we argue that patient-centeredness requires that the patient, if they are to participate meaningfully in the decision-making, is able to make their own trade-offs, by using their personal utilities. As a result, they may, for example, evaluate a designated second or third-line option (such as surgery) as superior to the “normal” first-line one (such as chemotherapy).

However, all the foregoing assumes that it is feasible for the personal utility set for the patient to be generated within the clinical consultation, not, as with the development of public sets, in a personally irrelevant research exercise*.* Moreover, for the utilities in that set to be used in the consultation as a means to enhance patient participation in, and co-production of, the decision. Otherwise, elicitation would be purposeless. This study presents the conceptual prototype needed as the analytical basis for a clinical trial protocol.

### The Clinical Potential of a New Approach

We have modified the novel online elicitation of personal utility functions (OPUF) approach to the development of a public utility set [2-4]. The novelty of OPUF arises from the fact that it achieves the public set of average utilities for each descriptive health state by aggregating the full utility sets of a sample of individuals. This contrasts with conventional methods, such as time trade-off and discrete choice experiment (DCE). In those, subsets of citizens choose between different samples of health state pairs, and the public set is arrived at by statistical inference. Their full personal utility set is never generated for any one individual.

While OPUF develops the personal utility sets for an individual in order to produce a public set, the developers are aware that the method “could potentially be useful for other applications beyond health economics… For example, the OPUF approach could be used as a patient decision aid and to facilitate shared decision making in a clinical context. Explicitly weighing different aspects of health might help patients, who face complex treatment decisions, to better understand the trade-offs that are involved, and what aspects are most important to them” [2].

This clinical potential was also recognized by the developers of another tool that generates public sets on the basis of a sample of full individual ones. “The PAPRIKA tool could also support Cost Utility Analysis and Patient Reported Outcome Measures at the individual patient level, incorporating the patient’s preferences into treatment decisions in “real time.” For example, the tool could be available on computer tablets in doctor waiting rooms or as a mobile app for patients to quickly create their own personal value sets [5].” PAPRIKA (Potentially All Pairwise Rankings of All Possible Alternatives) unfortunately fails our clinical feasibility test in requiring the patient to make a minimum of 20 pairwise comparisons, and 55 to properly generate the full OPUF utility set. The developers actually acknowledge the problem. “Notwithstanding the relative simplicity of PAPRIKA’s questions, almost half of the high-quality by education sub-sample reported finding them difficult to answer. This finding is unsurprising given the questions involve confronting trade-offs between EQ-5D-5L dimensions, which is unlikely to be a familiar cognitive task for most people” [5]. Hence, we make no further reference to this instrument (it also uses commercial software).

We develop this clinical potential, introducing the prototype of an instrument akin to the OPUF approach in producing a complete Personal Utility Set for the individual involved in a Healthcare decision (PUSH). However, PUSH is designed exclusively for use by clinicians and individuals in the context of a clinical decision, rather than by researchers surveying anonymous individuals online to produce a public utility set to support later and impersonal public (or group) decisions. There are major implications of this completely different real-time clinical decision support focus, notably the importance of adopting a contextual normativity that reflects both the ‘Time Needed to Treat’ [6] and the cognitive resources and capabilities of the two parties in the clinical encounter.

We follow OPUF in using the EQ-5D-5L framework for PUSH, though the approach is relevant to any multidimension, multilevel instrument in clinical use, including condition-specific ones. In relation to all such measures, we emphasize that the clinical presentation of utilities as percentages—or values to two decimal places—is not intended to imply this degree of measurement accuracy, rather to encourage and enable analysis-supported consideration of the relative magnitude of key parameters in the clinical context.

The following introduction to EQ-5D-5L incorporates some abbreviations to reduce prolixity.

### EQ-5D-5L

In the EQ-5D-5L framework, a person is described as being in one of five levels on each of five dimensions and therefore is in one of 5^5^ or 3125 unique health states. The 5 dimensions are mobility (MO), SC, UA, PD, and AD. They are always referred to in this order. The five levels are 1=no problems, 2=slight problems, 3=moderate problems, 4=severe problems, and 5=extreme problems. This means the best possible health state is described as 11111 and the worst possible as 55555. If the patient’s current health state is No MO, Moderate SC, Slight UA, Slight PD, and Severe AD, its descriptive summary code is 13224. Note that while these numbers have evaluative connotations in indicating an ordinal ranking on each dimension, those connotations are not part of the descriptive framework.

If an intervention (including no intervention) is contemplated, the described current and future possible health states have to be evaluated in order to establish how much gain (or loss) is estimated to result from undertaking it. So, if intervention X is estimated to move the patient from 13224 to 13332, they need to know how much better or worse the latter is than the former (from their personal perspective), given that their AD would be improved from Severe to Slight, but their PD and UA would worsen from Slight to Moderate (MO and SC unchanged). Note that ‘evaluating’ the individual health states (ie, assigning a utility to health state 13224 and to health state 13332) is not the same as ‘evaluating’ the intervention, which concerns the difference between the utilities placed on 13224 and 13332.

### OPUF

A demo version of the OPUF tool is available online [7]. Engaging with it is encouraged, but we extract the relevant key steps here, renumber them, and reword the instructions to the patient. It is important to register the cognitive demands of the process since these are the reasons for our clinical simplifications in PUSH. Some relevant comments from the OPUF UK study are added.

#### Step 1. Weigh the Health State Levels

Please place “slight,” “moderate,” and “severe health problems” on the Visual Analog Scale (VAS), which runs between 0% (“Extreme problems”) and 100% (“No problems”).

#### Step 2. Rank the Dimensions

Please rank the five dimensions (MO, SC, UA, PD, and AD) in terms of how bad their “extreme” (ie, worst) levels would be for you. 1=worst to 5=least worst. No ties permitted.

#### Step 3. “Swing Weight” the Dimensions

The slider for the dimension ranked 1 is fixed at 100 to indicate the value you would place on moving from Extreme to No problems on it. Use the other sliders to indicate the value, relative to 100, you would place on moving from Extreme to None on each of the other four dimensions.” The responses are normalized to add to 100% so as to produce the dimension weights.

“Criteria/dimension weights… represent the relative importance of a given criterion, compared to all other criteria. More specifically, it is a measure of the relative (utility) gain associated with replacing the lowest level with the highest level of performance for this criterion (eg, moving from extreme pain to no pain). A value of 100 is assigned to the most important criterion, and the weights of all other criteria are then defined relative to this yardstick: a value of 50, for example, means a criterion is half as important; a value of zero means a criterion is not important at all [2].”

#### Step 4. Evaluate the State “Being Dead”

Different methods are used depending on whether 55555 is preferred to being dead or vice versa.

If (A) you prefer being in health state “55555” to “being dead,” please place “55555” on a VAS running from “No health problems” (=100) to ‘being dead’ (=0) (you may place it at 0).

If (B) you prefer “being dead” to being in a health state “55555,” please respond to the following series of questions that will establish the “least worst” 5-dimension health state that you regard as equivalent to being dead. These are 6 adaptive DCE questions which involve choosing the preferred state from a pair of 5-dimensional ones (such as 13224 and 13332).

#### Step 5. Derive the Dimension-Level Coefficients

The level weightings are multiplied by the dimension weights to form a 5 by 5 disutility matrix (disutility equals decrement from full health). This is then rescaled to set the state regarded as equivalent to being dead equal to 0, with states valued worse than being dead as <0.

Two key points from the OPUF UK study:

1. “On average, it took participants about 9 minutes to complete the survey. The median was eight; the shortest duration was three; and the longest was 32 minutes [2].”
2. “We found that health state preferences systematically differed between groups... for age, having children, importance of religion/spirituality, and the EQ Visual Analog Scale quintile. However, the variability of preferences within groups was substantial, and... when all characteristics were taken into account together, group membership accounted for just 8% of the variance...The results illustrate that aggregate group‐level value sets usually say little about the preferences of any given individual – in our study, preferences differed greatly between individuals within all the groups that we considered [2].”

### PUSH: A Personal Utility Set for Health Care

The OPUF developers suggest that its time demand was not a major problem for their tool, though, from our own personal engagement with it, we believe the reported medians reflect thinking and response times that would be significantly increased in the clinical context with a real patient. Our approach implicitly addresses this time issue by reducing the task demands on the patient, but this is primarily to address the cognitive difficulties of swing weighting (step 3) and choice-based pairwise comparisons (step 4).

In introducing the aspects of PUSH that enhance its clinical appropriateness and practicality, relative to OPUF, we stress that it is vital that these are not seen as reluctant deviations from the research ideal. Normativity is contextual and therefore preference-sensitive, so if there is any “gold standard” by which to assess a clinical evaluation process, it has to be one defined with reference to clinical practice, not some research-appropriate but clinically irrelevant ideal (there cannot be a “gold standard” for an individual patient’s utility set elicited on a specific occasion). Moreover, we must avoid the partial or noncomparative evaluation of processes [8]. Evaluation of any evaluation process should involve explicit and empirical comparison with the actual “usual”—in this case, how a patient’s evaluation of the relevant treatment outcomes is actually undertaken—not with an implicitly assumed standards-compliant usual. Even accepting this, the problem of establishing what ‘actual usual care’ is, in any jurisdiction, remains formidable [9,10].

PUSH adapts OPUF in several ways to improve its clinical feasibility. It follows OPUF in eliciting and using the same level weights for all 5 dimensions, as opposed to seeking dimension-specific ones (like the OPUF developers, we are aware of the limitations of this assumption). However, to establish the patient’s dimension weights, it rejects the complex “swing weighting” procedure and simply elicits their relative importance in avoiding the worst outcome on each.

Furthermore, PUSH avoids the choice*-*based elicitation procedure that requires the person to make pairwise comparisons of multidimensional health states. We fully realize this is in direct contravention of the convention in public value set elicitation for clinical policy decisions, such as those implementing cost-effectiveness restrictions. “… The National Institute for Health and Care Excellence (NICE) requires utilities to be based on “choice-based” methods. time trade-off and DCEs are generally accepted as being choice-based; the Location of Death approach is also based on choice-based tasks.... VAS has tended to be rejected by health economists (with rare exceptions) on the grounds that it is not choice-based [11].”

Our preference for scale-based methods in PUSH partly reflects past unsatisfactory attempts to implement choice-based methods in a clinical setting [12,13], but is endorsed by Aström et al [14] in their recent scoping review of the use of the Visual Analog Scale (VAS) in health care.

“Compared to SG and TTO, the VAS was perceived as an easier and more practical approach. Consequently, using a VAS was associated with lower respondent burden and administration costs, and fewer measurement errors. The VAS was seen as more sensitive or discriminatory to symptoms and more culturally acceptable. The VAS was observed to have a better model fit and a similar predictive ability compared to the TTO... The most common criticism was that the VAS does not measure utilities...under uncertainty... However, Parkin and Devlin [15] argue that using the VAS involves both choice and trade-off across sets of health states, and that other methods also suffer from biases or concerns regarding generating reliable preferences” [14].

Finally, PUSH follows OPUF in not setting “dead” as the state with the lowest utility and equal to 0, this being incompatible with QALY evaluations for well-elaborated reasons [16]. However, to avoid the choice-based method of OPUF used only for individuals who prefer being dead to 55555 (B in step 4 above), PUSH diverges in how the particular patient locates the state they personally regard as being neither better nor worse than being “dead.” (The precise method is outlined in the following section.) It may be asked why, in the clinical setting, we need the patient to locate this state and have it assigned a utility of 0 on their personal utility scale, when the public set reason for this “anchoring” does not apply. The answer is simple, but quite profound. A patient’s whole personal utility set is absolutely higher, the stronger their expressed preference for their worst possible living state (55555 in EQ-5D-5L) over being dead. Conversely, their whole personal set is absolutely lower, the stronger the expressed preference for being dead over being in 55555. In other words, where the person locates the state regarded as equivalent to being dead, in relation to being in the worst possible living state, determines their absolute HRQoL. The strength of their preference for not being dead determines how healthy they are, according to their own values*.*

This may immediately make intuitive sense, but if not, an illustration might help. If one locates 55555 at, say, +30 (where being dead=0), all the more preferable states will have utilities concentrated in the range from +30 to +100 (where +100 is full health). The values for all these states will be absolutely higher than if they had located 55555 at –30 (when being dead=0), where they will also be dispersed over the much larger range from –30 to +100. A fuller patient example is provided in the following section.

### PUSH: Establishing the Patient’s Utility Set

PUSH for EQ-5D-5L is an Excel workbook, the spreadsheet platform being adopted to maximize accessibility and minimize the funding and maintenance requirements of bespoke programs. It is available on request to the corresponding author under the Creative Commons license CC BY-NC-SA. Columns A-H of sheet 1 constitute the user interface. Columns I-R are the hidden ‘back end’ that the clinician can unhide if interested. Other tabbed sheets contain the dropdown lists, country public utility sets, and sources.

It is envisaged that the clinician holds it as a template on their computer and opens a new copy for use with each patient. They agree with the patient on what, if anything, is to be saved as part of their electronic medical record.

As the first part of the descriptive phase of engagement, the patient rates their overall health today on the 0-100 scale of the EuroQol “thermometer,” where 0 is my “worst imaginable health state” and 100 is my “worst imaginable health state.” This is a EuroQol requirement for the use of EQ-5D-5L. We make no use of it in PUSH, but it is visibly available for use if desired.

The patient then responds to EQ-5D-5L, selecting the severity of their problems on its 5 dimensions. As in OPUF, these levels are automatically rephrased to the common set for all dimensions of no, slight, moderate, severe, and extreme problems. In our patient example, we will assume this procedure results in them reporting no MO problems, moderate SC problems, slight UA and PD problems, and severe AD problems. Their EQ-5D-5L health state is therefore summarily coded as 13224.

The evaluative section of PUSH now establishes the patient’s initial “unanchored” disutility matrix. The disutilities are the decrements in value from not being at Level 1 (no problems). The illustrative matrix in Table 1 is for Schneider’s exemplar: a person who located slight problems at 90 (disutility 10), moderate problems at 50 (disutility 50), and severe problems at 30 (disutility 70) on a scale where no problems are 100 (disutility 0) and extreme problems are 0 (disutility 100). These are inverted and decimalized to give their five personal level weights of 0, 0.1, 0.5, 0.7, and 1. The person’s responses on the dimension items, with their normalized weights in brackets, were MO 100 (.29), SC 60 (.17), UA 45 (.11), PD 80 (.23), and AD 70 (.20). Note that these dimension weights form the extreme problems row of the disutility matrix. Rows 1- 4 are created by multiplying the extreme dimension weight by the level weights. For example, the disutility for severe PD problems is .23* 0.7=.16, and that for slight SC problems is .17*0.1 = .02. The final column contains the health state utility when all five dimensions are at the weight level. Logically, since its 5 disutilities must add to 1, the utility of 55555 is 0.

**Table 1:** Unanchored OPUF disutility matrix for exemplar patient [2]. Utility of 55555=0.

Level	Weight	Mobility	Self-care	Usual activities	Pain/discomfort	Anxiety/depression	Utility
1: No problems	0	0	0	0	0	0	1
2: Slight problems	0.1	.03	.02	.02	.02	.02	.90
3: Moderate problems	0.5	.14	.09	.06	.11	.10	.50
4: Severe problems	0.7	.20	.12	.08	.16	.14	.30
5: Extreme problems	1	.29	.17	.11	.23	.20	0

OPUF’s adaptive pairwise questioning identified health state 51255 as being the one closest to being dead – neither better nor worse – for this person. Using the “unanchored” matrix above the disutility of 51255 emerges as .74 [.29 + 0 + .02 + .23 + .20]. To derive this patient’s “anchored” matrix in which 51255 has a utility of 0 (= being dead), OPUF therefore divides all the disutilities by .74 (Table 2). For example, Severe PD becomes .16/.74 or .22.

**Table 2:** Anchored OPUF disutility matrix for the patient. disutilities divided by .74.

Level	Weight	Mobility	Self-care	Usual activities	Pain/discomfort	Anxiety/depression	Utility
1: No problems	0	0	0	0	0	0	1
2: Slight problems	0.1	.04	.02	.02	.03	.03	.86
3: Moderate problems	0.5	.20	.12	.08	.16	.14	.30
4: Severe problems	0.7	.29	.16	.11	.22	.19	.03
5: Extreme problems	1	.39	.23	.15	.31	.27	–.35

Extreme health state 55555 now has a disutility of 1.35 [.39 + .23 + .15 + .31 + .27], and hence a utility value of 1-1.35, or–.35. 44444 has a utility of .03, 33333 one of .30, and 22222 one of .86 (rounding effects occur in some tables).

As a result of ruling out choice tasks, PUSH takes a scalar approach to the necessary anchoring (this means the OPUF distinction in step 4 vanishes). The patient is asked for their strength of preference in relation to being in health state 55555 and being dead, on a 7-point scale running from +30 (“Very strongly prefer 55555 to being dead” ) to –30 (“Very strongly prefer being dead to 55555”), with 0 representing “Indifferent between being in 55555 and being dead.” The resulting anchoring dividers (Table 3) for each of the possible patient responses ensure the utility of the state they regard as neither better nor worse than “being dead” is 0. Note that if the individual prefers 55555 to being dead, their utility for it will be above 0, just as it will be below 0 if they prefer being dead to 55555. Adopted for practice-practical reasons, this procedure does require the scale endpoints to be defined independently of the patient, though the particular ones in this illustration (+30 and –30) are not essential to the PUSH concept. The chosen ones effectively censor the 55555 “pits” utility at –.43, a value similar to that in many countries’ public tariffs and close to the –.35 of the Schneider exemplar patient.

**Table 3:** Rescaling anchors to set the utility of being dead to 0.

Preference for being in State 55555 vs being Dead	Select	Anchor
Very strongly prefer being in 55555 to being dead	30	1.3
Strongly prefer being in 55555 to being dead	20	1.2
Slightly prefer being in 55555 to being dead	10	1.1
Indifferent between being in 55555 and being dead	0	1
Slightly prefer being dead to being in 55555	–10	.9
Strongly prefer being dead to being in 55555	–20	.8
Very strongly prefer being dead to being in 55555	–30	.7

That this procedure closely replicates (Table 4) Schneider’s when –30 (“Very strongly prefer being dead to being in 55555”) is selected, is not surprising, since the .7 used as the anchoring divider is very near to .74. Extreme state 55555 is valued at -.43 compared with –.35 and 51255 is a few points below Schneider’s zero at –.05 [1 – (.41 + 0 + .02 + .33 + .29)]. The state with all Severe Problems (44444) emerges with the value of zero, equivalent to no better or no worse than being dead for this patient. However, given the PUSH elicitation procedure, it is not necessarily the only state with that value.

**Table 4:** Anchored PUSH disutility matrix for patient with .7 (not .74) used as an anchoring divider.

Level	Weight	Mobility	Self-care	Usual activities	Pain/discomfort	Anxiety/depression	Utility
1: No problems	0	0	0	0	0	0	1
2: Slight problems	0.1	.04	.02	.02	.03	.03	.86
3: Moderate problems	0.5	.20	.12	.11	.16	.14	.30
4: Severe problems	0.7	.29	.17	.08	.23	.20	0
5: Extreme problems	1	.41	.24	.16	.33	.29	–.43

### PUSH for EQ-5D-5L as Clinical Decision Support

The purpose of eliciting the patient’s complete value set in a clinical decision-making context is to facilitate their more effective participation by enabling their personalized evaluation of the individualized effects of interventions. Some conversation will always have occurred before the patient is asked whether they wish to engage with the PUSH decision support tool, and they will have given their informed consent. In our hypothetical illustration, we assume that, in response to a “what brings you here today?” type opening, the patient has reported feeling significantly depressed for some months and answered affirmatively to the 2 standard Whooley questions that guidelines recommend in this case [17]. This accounts for the 4 (severe AD) when their current state is summarized as 13224.

Following the generation of the patient’s value set, the clinician makes a set of predictive judgments. Using the best available evidence and information, including that from further conversation with the patient, they enter the health state they judge, on the balance of probabilities, to be the most likely for each contemplated intervention at a chosen future time point agreed with the patient (say 3 months). In the current (but extendable) PUSH template, the clinician does this for No Intervention and for Interventions A and B. In the current example, where depression is the presenting condition, A might be, for example, a medication, and B some version of cognitive behavior therapy.

While challenging, making these judgments is well within the remit of the clinician. If they have some relevant guideline in mind, such as those from NICE, they will be aware of the routine disclaimer that heads all NICE guidance concerning the supremacy of clinical judgment. “When exercising their judgment, health professionals are expected to take this guidance fully into account, alongside the individual needs, preferences, and values of their patients. The application of the recommendations in this guidance is at the discretion of health professionals and their individual patients and does not override the responsibility of health care professionals to make decisions appropriate to the circumstances of the individual patient, in consultation with the patient and/or their carer or guardian” [17].

### PUSH: A Specific Patient Example

A PUSH-based example using the level and dimension weights of the Schneider exemplar applied to a patient with current state 13224 appears in a screenshot from the tool (Figure S1 in Multimedia Appendix 1).

The patient’s current health state of 13224 has a PUSH-generated utility of .63 [1 – (0 + .12 +.02 + .03 + .20)]. A time horizon of 3 months is chosen for initial evaluation of the options.

If there is no intervention, it is the clinician’s judgment that, on the balance of probabilities, their depression will deteriorate from severe to extreme, with other dimensions unaffected. This would lower their utility from .63 to. 54 of state 13225. [1 – (0 + .12 +.02 + .03 + .29)].

The specified medication is judged likely to move them to 13332 - the major improvement in AD is seen as likely to be offset by the deteriorations in PD and UA as a result of side effects, leaving them at .60 [1 – (0 + .12 +.08 + .16 + .03)]. This is better than no intervention, but slightly worse than their current state. On the other hand, while the cognitive behavior therapy intervention is judged likely to produce less improvement in AD, it would not involve any deterioration in PD or UA from their current levels. It is accordingly judged likely to move them to 13223 and hence to .69 [1 – (0 + .12 +.02 + .03 + .14)]—an improvement on their current state.

The PUSH participation and its outputs are to be viewed as inputs into deliberative co-productive decision-making, where both parties contribute significantly but in role-appropriate ways. One key responsibility of the clinician is to ensure that any assistance during the elicitation phase is nondirective as far as how terms are to be interpreted (eg, “severe” and “strongly”), emphasizing that it is the patient’s interpretation that is sought. A second is to dispel any “aura of exactness” or pseudo-precision that may be created by the use of precise percentages (values to two decimal places) and only central point estimates. They should make clear that they are intended to provide a useful, analytically-framed input into discussion, which is likely to include some sensitivity testing of the level weights, dimension weights, and preference regarding 55555 and dead. All changes are instantaneously implemented and visible.

It is worth repeating why we need the patient to locate the state seen as equivalent to being dead (and setting it to 0), even when no life-threatening option is involved in the current decision. The reason is that where they locate being dead determines the absolute value they place on any living health state, and therefore on any states that result from contemplated interventions. Take the patient in our Table 5 example. They “Very strongly preferred being dead to being in 55555” and, given their level and dimension weightings, this results in their current state of 13224 being valued at .63. Intervention B (to take it as an example) improves it to .69. However, if they had been “Indifferent between being in 55555 and being dead” they would value both the current and Intervention B states higher, at .74 and .78 respectively. Finally, if they had “Very strongly preferred being in 55555 to being dead,” they would value their current state at .80 and Intervention B at .83, which are respectively 27% and 20% higher than those with the extreme reverse preference.

**Table 5:** Effect of 55555—dead preference on the utility of states in the patient example.

Preference	Current	No Intervention	Intervention A	Intervention B
EQ-5D-5L health state	13224	13225	13332	13223
Very strongly prefer being in 55555 to being dead	.80	.75	.79	.83
Strongly prefer being in 55555 to being dead	.78	.73	.77	.82
Slightly prefer being in 55555 to being dead	.76	.71	.75	.80
Indifferent between being in 55555 and being dead	.74	.68	.72	.78
Slightly prefer being dead to being in 55555	.71	.64	.69	.76
Strongly prefer being dead to being in 55555	.68	.60	.65	.73
Very strongly prefer being dead to being in 55555	.63	.54	.60	.69

While the rankings of states are unaffected, it is clear – and hopefully makes intuitive sense on reflection – that the more preferable one regards the worst possible living health state to being dead, the healthier one regards oneself. A patient who strongly prefers being alive even in extreme ill health will also see smaller absolute differences between treatment options than a patient who regards extreme ill health as worse than death. The implications of this pure preference-sensitivity—exposed but not created by its quantification—can have a substantial impact on the subsequent decisional deliberation. It seems unlikely that an intervention which is thought likely to move one’s self-valued health from .80 to .83 will have the same appeal as one that improves it from .63 to .69, even after discounting for the “pseudo-precision.” Figure S1 in Multimedia Appendix 2 is a diagrammatic version of Table 5.

The PUSH-based evaluations are automatically compared with those using the conventional public utility set for the relevant jurisdiction by selecting it in the country cell. In Figure S1 in Multimedia Appendix 1, China is the one selected from the set of 13 countries currently provided in the template (others are easily added). As shown, the Chinese tariff fortuitously returns very similar values to the Schneider-based example. However, there is great heterogeneity across the country public sets, as is well-known and can be easily confirmed by a click of the country cell in PUSH.

PUSH is easily adapted to other descriptive systems. We have already developed functional versions for two instruments with OPUF-derived value sets: the EuroQol Health and Wellbeing 9 scale (EQ-HWB-9) [18-20] and the MO and Quality of Life Seven Dimension index (MobQoL-7D) [21,22]. The corresponding author can be contacted for further information.

## Discussion

There is a world of difference between clinical decision-making with a patient and online research involving individuals as the source of inputs into impersonal public decision-making. The application of many legitimate principles and standards for the latter, appropriately accepted in OPUF, is ruled out for PUSH. The challenge has been one of meeting principles and standards appropriate for one-off, point-in-time, clinical decisions, where research considerations, such as test-retest reliability 2 weeks apart, to name one, are explored in relation to OPUF [23], have no relevance in practice. Swing weighting and comparing pairs of 5-dimensional health states have both been rejected on the basis of their time and cognitive demands. There are many indisputably clinically relevant challenges within the OPUF approach, even after these modifications are accepted. The most significant way in which PUSH addresses these is that patient participation and engagement occur in the company of a clinician who is competent and responsible in the use of the tool, not least because they will be making predictive clinical judgments within it as part of the co-production process.

We have not yet undertaken any formal trialing of PUSH. As would be expected, we have engaged in informal testing with colleagues and friends in the course of development. However, the formal requirements for any piloting of a clinical innovation that transforms both patient participation and clinician contribution to such a major extent mean it is sensible to advance it for discussion as a prototype to ensure a trial protocol (including clinician tutoring) is conceptually sound.

## Appendix

Multimedia Appendix 1 Screenshot of PUSH showing completed example from text.

Multimedia Appendix 2 Effect of preference for health state 55555 versus being dead on the absolute utility of current health state and that from different interventions.

## Abbreviations

AD: anxiety/depression
EQ-HWB-9: EuroQol Health and Well-being 9 9-item scale
HRQoL: health-related quality of life
MO: mobility
MobQoL-7D: Mobility and Quality of Life Seven7-Dimension index
NICE: National Institute for Health and Care Excellence
OPUF: online elicitation of personal utility functions
PAPRIKA: Potentially All Pairwise Rankings Of of All Possible Alternatives
PD: pain/discomfort
PUSH: personal utility set for health care
QALY: quality-adjusted life year
SC: self-care
UA: usual activities

## Fundign

No external financial support or grants were received from any public, commercial, or not-for-profit entities for the research, authorship, or publication of this article.
