# Supplementary material for: Enhancing Patient Participation in Co-Productive Decision-Making With Personal Value Sets: Clinical Trial Prototype
Source: J Particip Med. 2026 Jun 16;18:e81623. doi: 10.2196/81623 (PMC13320008; doi:10.2196/81623)
Supplement: Multimedia Appendix 1 [file jopm_v18i1e81623_app1.docx]

Figure S1: Screenshot of PUSH showing completed example from text.
