# Supplementary material for: Enhancing Patient Participation in Co-Productive Decision-Making With Personal Value Sets: Clinical Trial Prototype
Source: J Particip Med. 2026 Jun 16;18:e81623. doi: 10.2196/81623 (PMC13320008; doi:10.2196/81623)

Figure S1. Effect of preference for health state 55555 versus being dead on the absolute utility of current health state and that from different interventions (Source: produced by Claude Sonnet 4.5 from Table 5 in text.)

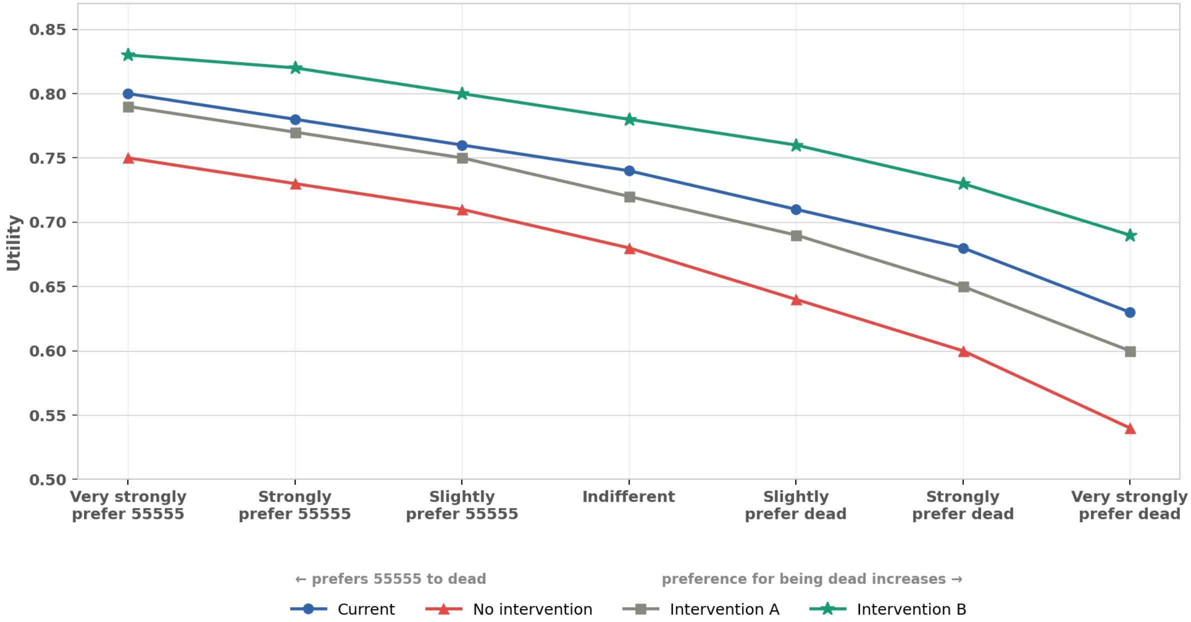

Supplement: Multimedia Appendix 2 [file jopm_v18i1e81623_app2.pdf]
